# Random Effects Models and Multistage Estimation Procedures for Statistical Population Reconstruction of Small Game Populations

**DOI:** 10.1371/journal.pone.0065244

**Published:** 2013-06-03

**Authors:** Christopher M. Gast, John R. Skalski, Jason L. Isabelle, Michael V. Clawson

**Affiliations:** 1 Quantitative Ecology and Resource Management, University of Washington, Seattle, Washington, United States of America; 2 School of Aquatic & Fishery Sciences, University of Washington, Seattle, Washington, United States of America; 3 Missouri Department of Conservation, Columbia, Missouri, United States of America; 4 School of Environmental and Forest Sciences, University of Washington, Seattle, Washington, United States of America; National Institute of Environmental and Health Sciences, United States of America

## Abstract

Recently, statistical population models using age-at-harvest data have seen increasing use for monitoring of harvested wildlife populations. Even more recently, detailed evaluation of model performance for long-lived, large game animals indicated that the use of random effects to incorporate unmeasured environmental variation, as well as second-stage Horvitz-Thompson-type estimators of abundance, provided more reliable estimates of total abundance than previous models. We adapt this new modeling framework to small game, age-at-harvest models with only young-of-the-year and adult age classes. Our Monte Carlo simulation results indicate superior model performance for the new modeling framework, evidenced by lower bias and proper confidence interval coverage. We apply this method to male wild turkey harvest in the East Ozarks turkey productivity region, Missouri, USA, where statistical population reconstruction indicates a relatively stationary population for 1996–2010.

## Introduction

Although the wildlife literature has seen models for age-at-harvest data in the past [1], [2], models for these types of data entered the forefront of monitoring population status and trends only recently [3]–[9]. Some of the most recent developments require estimation of initial animal cohort abundance as a parameter [3], [4], [7], [8], or as a latent variable [5] in a frequentist or Bayesian framework, respectively. Recently, models for statistical population reconstruction (SPR) of harvested large game animals have been developed that utilize the same likelihood-based inference techniques, but instead consider estimating animal abundance following optimization, outside of the likelihood framework with a Horvitz-Thompson-type estimator, which adjusts the observed harvest count by the estimated probability of harvest in accordance with the assumption of a binomial sampling scheme [9]. These recent developments constitute improvements over previous models of this nature, particularly when stochastic environmental factors may affect population dynamics [9].

Statistical population reconstruction of small game populations, however, imposes additional challenges. Often, only two age classes are distinguishable from one another. Adults, which may be one year or older, typically cannot be distinguished from one another and only distinguishable from the young-of-the-year. The older two age classes, then, represent a combination of animals born in the previous year that survived to the current year, as well as the adults that survived an additional year or more following their first year. In comparison to fully aged harvests of many big game animals, this represents a considerable loss of cohort data upon which the SPR models of Gove et al. [3], Skalski et al. [4], Skalski et al. [8], and Gast et al. [9] rely. On the other hand, Skalski et al. [10] found little loss in precision when big game population reconstruction was based on pooling adult harvest information for age classes 3+.

Previous work has examined a modeling framework for small game animals that provides a way to accommodate the unknown contribution of prior cohorts to the adult age group of a given cohort [1], [7]. These models, however, suffer from similar difficulties associated with the big game models of Gove et al. [3], Skalski et al. [4], and Skalski et al. [8] inasmuch as they assume constancy of demographic rates across time periods when additional data are not available to inform estimation of year- or age-specific rates. In addition, models with only fixed effects have proven in fully aged simulation studies to provide biased estimates of annual abundance and poor confidence interval covariate [9]. It is therefore of interest to extend the modeling framework of Broms et al. [7] in a manner similar to Gast et al. [9], and to determine what impact this has on statistical population reconstructions of small game populations with pooled age classes.

## Materials and Methods

### Models for Statistical Population Reconstruction of Small Game Populations

Laake [1], using a model formulation for age-harvest data originally proposed by Dupont [2], proposed optimizing a likelihood via the expectation –maximization (EM) algorithm [11], wherein components of the age A+ category (the oldest distinguishable age) in a particular year that belong to different cohorts are included by weighting the expected catch for each cohort relative to the total expected catch of all contributing cohorts. These weights must be estimated and, of course, rely on the current parameter estimates. Therefore, the expected catch (being treated as observed data in the likelihood function) changes during the optimization phase. Optimization of the likelihood function proceeds as usual, using these expected values of catch produced by the weighting, until convergence is achieved.

An alternative method [7] involves writing a likelihood that is conditional on having been observed (harvested), and using the data and parameters to estimate the probability of having been harvested in a given year as the ratio of harvest in that year to total harvest of the cohort and previous cohorts contributing to the A+ category. To see this more clearly, consider a sample age-at-harvest matrix given in Table 1, and consider the case where animal age can only be determined as young-of-the-year (age ½ year at a fall harvest) and adult (age 1½ year at a fall harvest). The adult category for each year of data is then a composite of the prior year’s surviving juveniles plus the prior year’s surviving adults, indicated by the shaded age of prior years to the final highlighted cell, 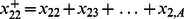, where the “+” in 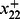 indicates it is a composite of all older age classes. Note that cohorts are numbered chronologically; upon collapsing adult age classes, *N*_1_ indicates the abundance of all adults upon initiation of the statistical population reconstruction, *N*_2_ represents the young-of-the-year in year 1, *N*_3_ represents the initial cohort (recruit) abundance in year 2, and so on.

**Table 1:** Example of age-at-harvest data.

*N_A_*	*N*_A-1_	*N*_A-2_	*N*_A-2_	*N*_A-3_	...	
*N*_A+1_	*x*_11_	*x*_12_	*x*_13_	*x*_14_	_…_	*x*_1,A_
*N*_A+2_	*x*_21_	*x*_22_	*x*_23_	*x*_24_	_…_	*x*_2,A_
*N*_A+3_	*x*_31_	*x*_32_	*x*_33_	*x*_34_	_…_	*x*_3,A_
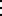	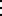	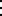	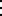	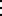	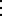	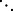
*N_A_*_+*Y*−1_	*x_Y_*_1_					

Consider the cohort corresponding to the main diagonal of Table 1. If we express the likelihood of the observed harvest as conditional on harvest, we may write the binomial likelihood as follows:
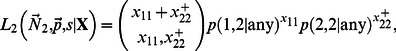
(1)where *x_ij_* denotes the number of animals in cohort *j* that are harvested in year*_i_*, and the notation 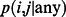 indicates the probability of a member of cohort *j* being harvested in year *_i_*, given that they were ever harvested. This binomial formulation requires the assumption of independent of fates of each animal within a given cohort and year.

A reasonable estimate for 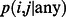 is the percent of total harvest observed in year *_i_* of cohort *j*. For example, the estimate of 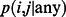 (cohort *j* = 2 begins with initial abundance *N_2_*) is
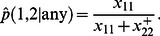
(2)


Standard maximum likelihood theory for multinomial models tell us this is, in fact, the maximum likelihood estimate for the probability of harvest in the *x*_11_ cell of the age-at-harvest table.

In order to rewrite the cohort likelihood in Equation (1) in terms of the parameters to be estimated, we replaced the observed harvest values in 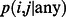 with their expected value, computed as a function of the parameter values. That is, instead of estimating 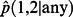 as in Equation (2), we instead used
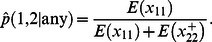
(3)


In this case, under an assumption that each harvest count is marginally binomially distributed, we computed each expected value as the number of animals from prior cohorts that survived the prior year’s harvest as well as the nonharvest season, and were subsequently harvested in the following year:
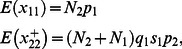
(4)where 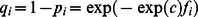 is the probability of surviving the harvest process in year *_i_* which is dependent on a harvest vulnerability parameter, *c*, and a measure of hunter effort in, and a measure of hunter effort in year *i*, *f_i_* [12] (pg. 296), and s*_i_* is the probability of surviving the nonharvest season immediately following the harvest season in year *i*. Similarly, we computed the expected harvest count for cell (3,2) of the age-at-harvest matrix as



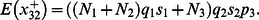



If sufficient auxiliary data are available, the probabilities may be made dependent on age class as well as year.

We computed each expected harvest count in this manner, and we created the joint likelihood of all cohorts as the product of individual cohort likelihoods. The likelihood contribution for cohorts 1 (the first observation of age class 2+) and 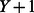 (the last observation of age class 1) cannot be included in the likelihood in the manner described above, so they are included as binomial components, such as
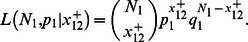



As in Gast et al. [9], we adopted the belief that unmeasured randomly distributed (and, in the work presented here, mutually independent) factors affect demographic processes temporally and, thus, we may include these random effects in the model formulation. To do so, we created the transformations
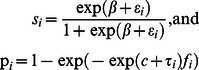
(5)and hypothesized that
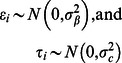
(6)and substituted the members of (5) into the appropriate locations in the likelihood function. In these formulations, the parameters 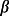 and c represent base conditions for survival and harvest vulnerability, respectively, that are modified by random effects. In general, these random effects need not be independent of one another or normally distributed. More complex formulations may be used by considering multivariate formulations for dependence between 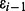 and 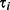, or within 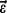 and/or 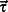. With the current formulation, we then must augment the joint likelihood with the normal distribution components to form the joint likelihood
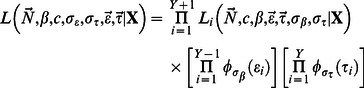
(7)where 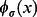 denotes the normal distribution density with mean 0 and variance 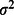.

The Horvitz-Thompson estimation approach may be employed to consider reduced-parameter models which avoid the direct estimation of initial cohort abundance via maximum likelihood in exchange for a second-stage abundance estimator based on adjusting the observed harvest count by the estimated harvest probability. This method of estimating abundance has proven successful in other fields [13], [14], and in models for fully aged datasets of large game [9]. In this case, we first computed the estimate of initial recruit abundance using the Horvitz-Thompson estimator.
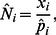
(8)where the current parameter values are used to compute 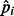. We simply proceeded to compute the cohort likelihood by first computing the expected harvest counts as in Equation (4).

Models for statistical population reconstruction based on age-at-harvest data require auxiliary data sources such that all parameters are identifiable [3]. For this reason, independent auxiliary data sources are sought to augment the age-at-harvest likelihood to provide parameter identifiability and estimability. Potential data sources include mark-recapture experiments, radiotelemetry studies, aerial surveys, and many other techniques common to the wildlife literature whose likelihood, denoted *L*_aux_, will share parameters with the age-at-harvest likelihood. The joint likelihood of the age-at-harvest and auxiliary likelihood is formed as the product of the two (or more) components, which is integrated over the random-effects terms to form the marginal likelihood of the model parameters,
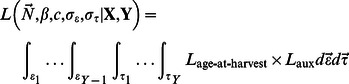
(9)where 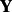 represents the auxiliary data source.

An auxiliary catch-effort likelihood which requires no additional data is formed by considering total annual harvest to be a binomial sample from the unknown total annual abundance 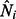 with the unknown harvest probability, 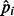, as in Skalski et al. [4] (Equation 3). The use or omission of this likelihood component constitutes a dimension of the study presented herein.

This marginal likelihood, integrated over the random-effects terms, is approximated with the Laplace approximation with Automatic Differentiation Model Building (ADMB) [15] software (the only such prepackaged software widely available for fitting nonlinear mixed-effects models of this nature in a frequentist framework) and optimized. Empirical Bayes estimates of the random effects 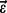 and 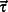 are available once maximum likelihood estimates of other model parameters are available. When the Horvitz-Thompson estimator is employed rather than direct estimation of each initial cohort abundance, the 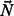 component is omitted from the likelihood in Equation (9), and the estimates of abundance required by the model are obtained with the Horvitz-Thompson-type abundance estimator during optimization. This method of first computing an estimated value of the variable 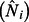 and then using it as data in the auxiliary likelihood takes the form of an EM algorithm.

Upon completion of likelihood optimization, we estimated standard errors from the inverse-Hessian matrix (for parameters) and the delta method (for functions of parameters). When the absolute quantity of each initial cohort abundance, 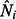, directly, we estimated the age-class abundance of the population by.
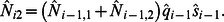



For the second-stage abundance estimation approach, we used the Horvitz-Thompson-type estimator of Equation (8) individually for each cell of the age-at-harvest matrix, 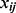, with its corresponding estimate of harvest probability, 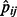, as 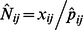. Uncertainty estimation for the Horvitz-Thompson abundance estimation approach requires an additional component associated with the second-stage estimator, which we described in the Appendix of Gast et al. [9].

We use the codes *HT_RE_* and *HT_FE_* to refer to models where abundance is estimated with a Horvitz-Thompson estimator using random effects (RE) and fixed effects (FE), respectively. The codes *AA_RE_* and *AA_FE_* are used to refer to models where the initial absolute abundance (AA) of each cohort is estimated directly, within the likelihood for random effects and fixed effects, respectively.

### Simulation Model

We conducted a simulation study to assess the ability of the models described above to produce successful estimates of population abundance and demographic parameters. Our simulation study involved simulating 1,000 populations with a chosen set of demographic parameters, along with random interannual effects, fitting each model to each dataset, and computing summary measures to describe how accurate and precise model-based estimates were in reconstructing population demographics.

We conducted simulations by first choosing a set of fixed demographic parameters (levels of 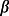, *c*, and reproduction) that led to a population of approximately 40,000 individuals with chosen characteristics (such as a population with abundance approximately stationary in expectation), creating an initial stationary population distribution, evolving this population with random interannual fluctuations included in the process parameters for 75 years, and then capturing the final 25 years as the harvest data to be used for reconstruction. We used a stochastic Leslie matrix [16] formulation to simulate animal populations with a single harvest vulnerability parameter (*c*) and a single survival probability parameter 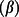 for all age classes. We assumed simulated variations in demographic processes (Table 2) would affect each age class similarly within a given year, and therefore 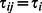 and 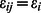, 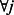. We simulated harvest counts and number surviving as binomial processes separately for each age class and each year, and we assumed harvest to be known precisely. We assumed recruit abundance to be `linearly dependent on the number of prior breeding-age adults, and this relationship was also assumed to be a Poisson process, which was also simulated with lognormal interannual variation in the stock-recruit parameter. We chose input parameters (Table 2) to produce a stable 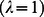 population at zero, low, medium, and high levels of simulated variation.

**Table 2:** Variation induced in natural demographic parameters of interest for small game simulation study.

Levels of stochasticity	Survival probability 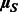 = 0.50	Annual harvest rate 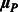 = 0.40	Fecundity 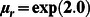 young/adult
Low	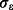 = 0.1 (0.450, 0.550)	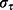 = 0.1 (0.338, 0.460)	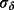 = 0.1 (6.050, 9.025)
Medium	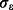 = 0.2 (0.401, 0.599)	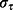 = 0.2 (0.287, 0.529)	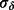 = 0.2 (4.953, 11.023)
High	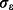 = 0.3 (0.354, 0.646)	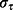 = 0.3 (0.242, 0.601)	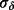 = 0.3 (4.055, 13.464)

(Harvest probability assessed at mean level of effort.).

Auxiliary data are required to fit the models to data. The amount of simulated auxiliary data available constituted another dimension for this simulation study. As small game animals are often easy to tag, we were interested to examine model performance under conditions when a relatively large amount of auxiliary data was available. For this reason, we conducted simulations with 6 years of radiotelemetry with 30 animals at risk annually that were used to aid in estimation of harvest probability. We were also interested to determine how sensitive the simulation results were to the amount of available data. For this reason, we conducted simulations with only a single year of radiotelemetry data comprised of 30 tagged animals. In both cases, the auxiliary data occurred in the middle of the 25 years of simulated age-at-harvest data (year 12 for the single-year case, years 9 through 14 when 6 years are available). In both cases, the number of animals detected as live following the harvest season and the known number tagged yields (by subtraction) a binomial sample from the number tagged with probability of harvest equal to the probability of harvest of untagged animals. These data were included in the joint likelihood as the product of 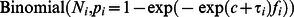 densities.

We examined the quality of reconstructions by comparing the median of the relative (percent) bias (MRB) in total abundance
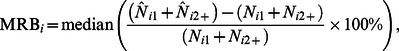
as well as the estimated confidence interval coverage of the 1,000 simulated samples provided by asymptotic 95% confidence intervals computed as



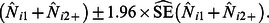



We estimated confidence interval coverage as the proportion of true annual abundances contained within the 95% confidence interval for each model, for each year of reconstruction, aggregated across simulations.

### Statistical Population Reconstruction for Wild Turkeys in Missouri, USA

In order to demonstrate the modeling procedure, we examined an age-at-harvest dataset of male wild turkeys (*Meleagris gallopavo*) collected from the East Ozarks turkey productivity region in the State of Missouri, USA (Table 3). The Ozarks East turkey productivity region consists of the following counties, which are located in southeastern Missouri: Butler, Carter, Crawford, Dent, Iron, Madison, Oregon, Reynolds, Ripley, St. Francois, Shannon, Washington, and Wayne. Nearly the entire 23,167 km^2^ region is located within the Ozark Highlands section. This heavily forested region is dominated by rugged terrain, which consists of highly dissected hills and streamside breaks [17]. Oak (*Quercus* spp.), hickory (*Carya* spp.), and shortleaf pine (*Pinus echinata*) are common in the Ozark forest [18]. Elevations within the region range from 85 to 540 m.

**Table 3:** Male wild turkey harvest data, Ozarks East turkey productivity region, Missouri, USA.

Year	Spring harvest juveniles	Spring harvest adults	Spring harvest effort	SE of effort	Poult/hen ratio	Fall archer index	Spring NPR juveniles	Spring NPR adults	Fall harvest juveniles	Fall harvest adults	RT at-risk juveniles	RT harvested juveniles	RT at-risk adults	RT harvested adults
1995	–	–	–	–	1.4	–	–	–	–	–	–	–	–	–
1996	626	2703	6.9298	0.519	2.1	364	53	279	253	208	23	0	32	7
1997	596	1764	6.5852	9,442	1.8	188	60	174	114	94	12	2	46	13
1998	847	2855	6.5854	0.384	3.1	541	102	255	414	115	4	2	16	8
1999	1833	1716	7.1273	0.401	2.1	263	185	197	149	154	30	3	9	5
2000	767	4237	6.2601	0.34	2.8	365	101	406	340	133	8	2	33	17
2001	1580	2781	7.6457	0.448	2.6	565	289	458	352	288				
2002	1388	3399	7.4921	0.462	1.3	359	286	591	201	236				
2003	884	4287	6.935	0.409	1.7	384	201	807	204	201				
2004	1046	3289	8.1414	0.984	2.0	521	195	600	292	295				
2005	887	2877	8.2299	0.527	1.5	224	345	799	215	198				
2006	829	3033	6.7334	0.431	2.2	294	311	823	349	232				
2007	904	2077	8.0017	0.483	1.2	342	349	691	381	504				
2008	501	3011	6.0511	0.381	1.3	207	244	899	162	146				
2009	791	2429	6.7649	0.394	1.6	326	268	692	309	276				
2010	840	2233	6.2808	0.344	1.1	161	337	762	176	164				

Effort = survey estimated hunter trips per 10,000. Effort SE = standard error of estimated hunter effort. Poult/hen ratio = survey estimated poult-per-hen count. Fall archer index = abundance index based on Ozarks East fall archer counts. NPR = non-permittee removal. RT = radiotelemetry.

Two harvests are conducted in this area annually, in spring and in fall. The larger spring harvest is of males only, while the smaller fall harvest is of both males and females. In addition, the spring hunt allows for unpermitted landowner harvest as well as a youth hunter season, while the fall hunt allows for unpermitted landowner harvest and an archery harvest (Table 3). Sex and age class (juvenile or adult) are determined for each harvested animal at hunter check stations, or more recently, by hunters themselves through an online check system. Because of the greater and better records of harvested males, we chose to perform a statistical population reconstruction of only the male component of the population. The period of data used for statistical population reconstruction extends from 1996 to 2010, with 5 years of auxiliary radiotelemetry data (1996 to 2000, Table 3). In addition, an independent estimate of the poult-to-hen ratio was available for each year of reconstruction, as well as an index of abundance arising from fall archery hunter counts (Table 3). Hunter effort information for the spring permitted hunt (Table 3) is presented as the number of hunter trips/10,000, estimated from a post-season survey.

A joint likelihood model of the multiple data sources was formed for the male turkey population. The primary likelihood component for the age-at-harvest data was formed, as described in Equations (7) and (9). Separate harvest vulnerability coefficients (*c_J_* for juveniles, *c_A_* for adults) were used to model separate harvest probabilities for juveniles and adults.

We assumed the auxiliary radiotelemetry harvest data to be binomially distributed from the number at risk, with probability of harvest equal to probability of harvest of unmarked animals. Therefore, the auxiliary likelihood component was included as.
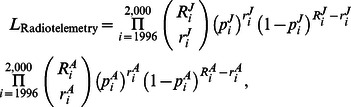
(10)where 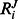 and 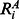 represent the known number of tagged individual juvenile and adult male turkeys, respectively, at risk in year *i*; 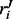 and 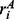 represent the known harvest count of the number of juveniles and adult males, respectively, at risk; and 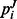 and 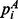 represent the harvest probabilities of juveniles and adults, respectively, in year *i*.

For both the age-at-harvest and auxiliary radiotelemetry data, harvest probability was parameterized as
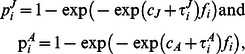
(11)where we assumed 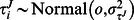 and 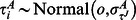.

No auxiliary information was available to support examination of separate natural survival probabilities for juveniles and adults, so we used a single survival parameter,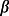, for both age classes. As there are two survival periods (the period between spring and fall harvest of year *i* [22 weeks, summer], and the period between fall harvest in year *_i_* and spring harvest of year *i*+1 [24 weeks, winter]), we assumed that the survival rate 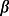 was constant across time within a year, but that interannual variation is present across years. Survival probability was therefore parameterized as
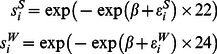
(12)to maintain bounding between (0,1), as well as to make survival probability dependent on the length of the interval the turkey must survive, in weeks(S = summer, W = winter). It was assumed that 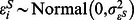 and 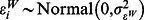.

**Figure 1:**
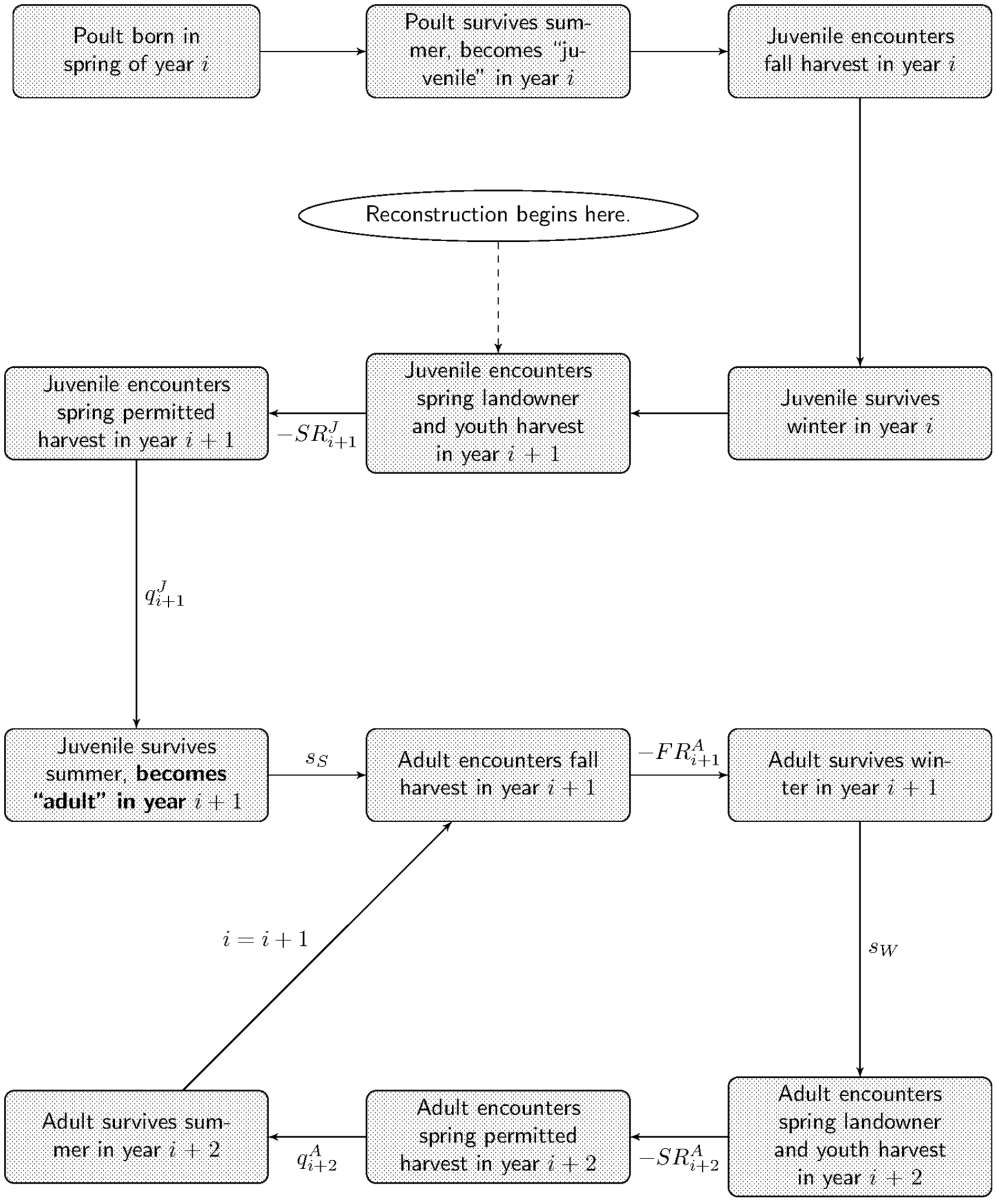
Turkey life-history flowchart. Death is not explicit but can occur at many possible nodes (natural mortality in summer or winter, harvest in spring or fall).



Modifications to the age-at-harvest likelihood were necessary to account for animals taken outside of the general spring permitted harvest, which were considered known removals for this analysis, because 1) no measure of effort is available for these harvests or 2) there was insufficient auxiliary or harvest data. We assumed, for purposes of the model, that spring known removals occurred prior to the permitted spring harvest season. We also assumed the turkey population to have the life history as detailed in Figure 1. Therefore within our model, in order for juveniles (age >9 months) in year *i* to be available for harvest as adults (age >1 year) in year 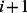, they must not be removed in the spring landowner or youth harvest and survive the spring permitted harvest. After the spring permitted harvest (when turkey are greater than age 1 year), they are classified as adults, where they then must survive until the fall harvest, survive the fall harvest, and survive until just prior to the following year’s spring landowner removal period. As adults may survive more than 1 year, there is a possibility for older adults to stay in the adult age class, rather than being removed from the population. Therefore, the expected number of adults available for harvest in year 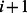 is

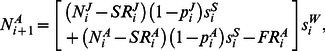
(13)where 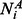 = adult abundance in year *i,* 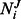 = juvenile abundance in year *i*, 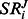 = juvenile spring removals in year *i*, 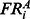 = adult fall removals in year *i*.

Note that the number of juveniles removed in fall does not enter the equation, because fall juvenile removals are confounded with the number of male poults produced, and the number of these that survive the summer. Since an estimate of this survival probability (and data that might be used to estimate this survival probability) is not available, an estimate of poult abundance is not available. Abundance of juveniles in year *i*, then, is the abundance immediately prior to the spring juvenile removal, when the turkeys are roughly 1 year old and have already undergone a fall harvest at age 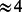 months.

We estimated annual abundance as the total male abundance prior to the spring harvest season. Therefore, in the age-at-harvest likelihood component, annual abundance is estimated as
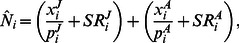
(14)such that the Horvitz-Thompson estimator is used to scale up the harvest counts (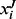 and 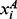 for juveniles and adults, respectively) via the probability of harvest, and the spring landowner removals are added to this to obtain the total estimated number of turkeys in the spring of year *i*. Fall harvest is considered a known removal in this model (because there is insufficient data across all years of the study), therefore the estimated count cannot be written as a function of model parameters. The consequence of this is that variability in annual abundance will be slightly underestimated, because the variability associated with the fall juvenile male removal is not accounted for.

The joint likelihood is written as the product of the age-at-harvest likelihood, the radiotelemetry likelihood, and the normal density components associated with the random effects 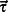 and 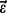. The marginal likelihood, integrated over the random-effects components, is approximated with the Laplace approximation, which is then optimized numerically with ADMB [15] software.

## Results

### Simulation Results

We omitted model *AA_FE_* from results that do not include the auxiliary catch-effort likelihood of Skalski et al. [4] (Equation 3) due to numerical instability during optimization. The absolute-abundance models depend heavily on the cohort structure of the age-at-harvest likelihood, and the catch-effort auxiliary likelihood helps support that structure. However, Horvitz-Thompson type estimators (i.e., Equation 8) are first-order, unbiased, without the need for any information on cohort structure.

**Figure 2:**
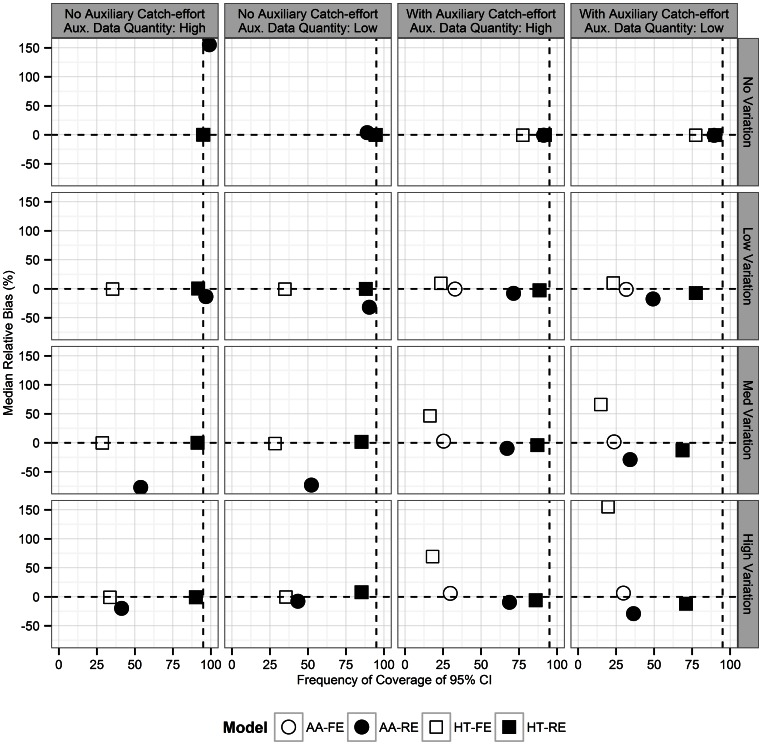
Monte Carlo simulation study results. Median relative bias and estimated coverage of asymptotic 95% confidence intervals for estimated total annual abundance for simulated small game data. Each point represents the mean of the median over 1000 iterations of 25 years of simulated data. Results indicate lowest bias and optimal coverage for models employing the Horvitz-Thompson abundance estimator and random effects.

Median relative bias of total annual abundance based on 1,000 simulations was lowest for random-effects models employing the Horvitz-Thompson (*HT_RE_*) abundance estimator (Figure 2), particularly when the auxiliary catch-effort likelihood of Skalski et al. [4] (Equation 3) is omitted. This model, *HT_RE_*, shows bias near 0% regardless of the level of simulated variation (rows of Figure 2) or quantity of auxiliary data simulated (two leftmost columns of Figure 2). The corresponding fixed-effects model, *HT_FE_*, also showed low bias (results overlapped with those of *HT_RE_* for the case when no interannual variation was simulated in demographic processes). The random-effects version of this model showed nearest-to-nominal confidence interval coverage of the asymptotic 95% confidence intervals, with coverage ranging from approximately 84% (when a high magnitude of interannual variation was simulated) to 96% (when no interannual variation was simulated, and a low amount of auxiliary data was available).

In contrast, the fixed-effects model of Broms et al. [7] that estimated each initial cohort abundance as an individual parameter, *AA_FE_* (which could only be fitted when the auxiliary catch-effort likelihood of Skalski et al. [4] (Equation 3) was incorporated) exhibited low bias, but very poor estimated confidence interval coverage, which dipped as low as 22% and was less than 40% in all cases of nonzero simulated environmental variation. The modification of this model to incorporate random effects, *AA_RE_*, produced estimates of total annual abundance that were negatively biased (except for the case of no simulated interannual variation), with accompanying subnominal confidence interval coverage estimates, except for the case of low simulated variation when the auxiliary catch-effort likelihood was omitted.

In the base-case scenario, when no interannual variation was present, all models showed some ability to accurately and precisely reconstruct total annual abundance, provided the correct choice of the use of the auxiliary catch-effort likelihood of Skalski et al. [4] (Equation 3) was made. The model that worked best across all simulated scenarios was model *HT_RE_*.

**Figure 3:**
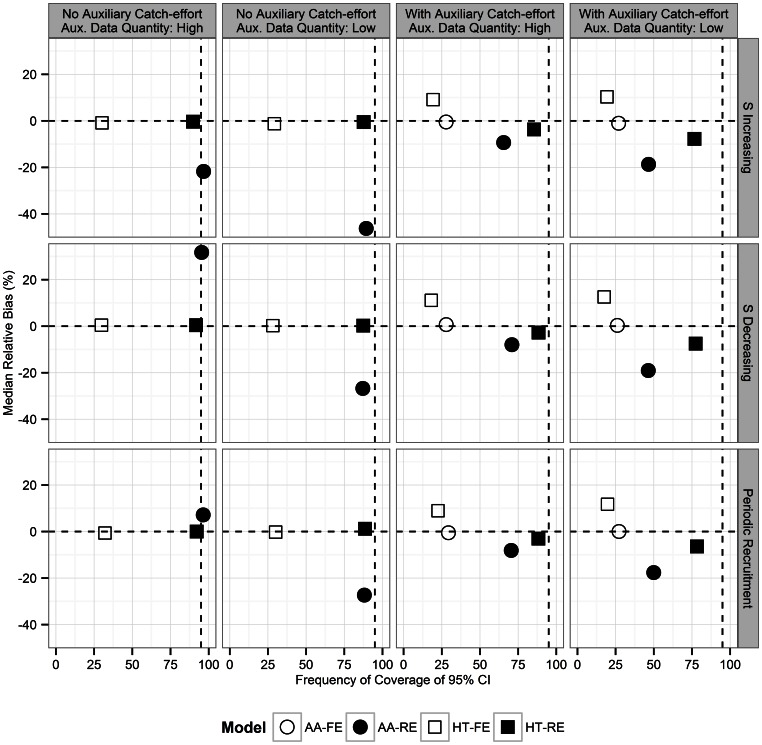
Monte Carlo simulation study results examining model robustness. Median relative bias and estimated coverage of asymptotic 95% confidence intervals for estimated total annual abundance for simulated small game data from simulations designed to address robustness. “Increasing S” (“Decreasing S”) scenario corresponds to a population with natural survival probability gradually increasing (decreasing) over the study period. “Periodic Recruitment” corresponds to a population with periodic positive and negative fluctuations in the annual recruitment rate, in addition to simulated natural interannual variability. Each point represents the mean of the median over 1000 iterations of 25 years of simulated data. Results indicate lowest bias and optimal coverage for models employing the Horvitz-Thompson abundance estimator and random effects.

In addition to simulations where model assumptions were valid, additional robustness simulations were conducted to assess the quality of statistical population reconstruction when some model assumptions were not satisfied. These scenarios were 1) the “Increasing S” scenario, where natural survival probability exhibits an increasing trend over the course of the reconstruction, 2) a “Decreasing S” scenario, where natural survival probability gradually declines, and 3) a scenario where overall population abundance is relatively flat (on average) but there were periodic pulses of births every fourth year (years 4, 8, 12, etc.) with accompanying crashes in recruitment rate in alternative four-year increments (years 2, 6, 10, etc.). All of these simulations were created with the low level of simulated variability (Table 2). The overall model performance across these three additional scenarios (Figure 3) was similar to the previous simulation results when all model assumptions were satisfied (Figure 2); the random-effects model employing the Horvitz-Thompson estimation approach provided nearly-unbiased estimates and near-nominal interval coverage, provided the auxiliary catch-effort likelihood component of Skalski et al. [4] (Equation 3) was omitted. The three other alternatives suffered from subnominal confidence interval coverage and/or biased estimates of total annual abundance.

### Missouri Wild Turkey Reconstruction Results

Based on the results of the simulation study, we fitted model *HT_RE_* to the Missouri male wild turkey data. Comparisons of Akaike’s information criterion [19], [20] using the conditional likelihood indicated the best model is that which uses two harvest vulnerability coefficients (*c_J_* and *c_A_*) and a single natural survival parameter. Subsequent model selection results for random effects using likelihood ratio tests for bounded parameters [21], [22] based on the conditional likelihood indicated separate random-effects terms for each age class (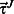 and 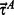), and no random effects for natural survival 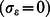 was the optimal model.

**Figure 4:**
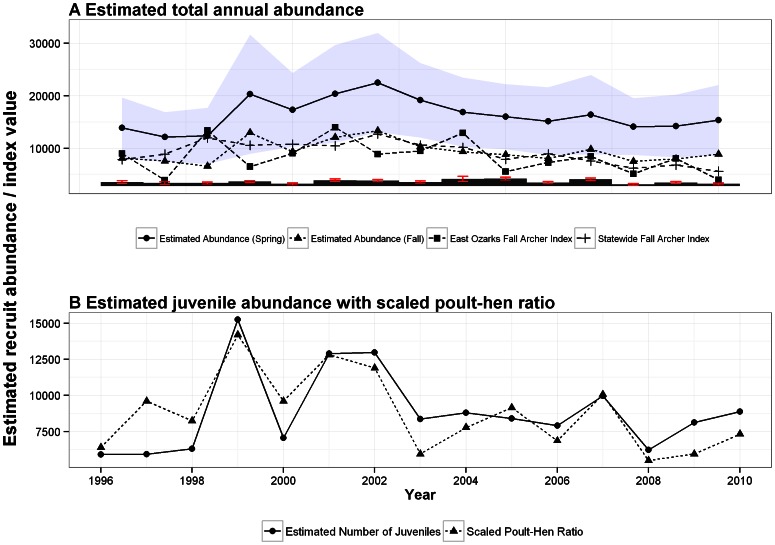
Estimates arising from the turkey population reconstruction. a) Estimated total annual abundance of juvenile and adult male wild turkey, Ozarks East turkey productivity region, for both spring and fall (with a confidence interval band for the spring abundance). Shown also is the Ozarks East fall archer count abundance index, which has been linearly rescaled to be shown on the same axis as estimated fall abundance. Linearly rescaled hunter effort is presented as vertical bars along the *x*-axis, with accompanying error bars indicating estimated effort ± SE. b) Estimated juvenile abundance with rescaled hunter-estimated poult-to-hen ratio. The poult-to-hen ratio has been advanced one year to align it with the estimated number of juveniles produced within each cohort.

Spring abundance estimates of juvenile and adult male turkeys indicated the population numbered approximately 13,875 individuals (95% CI: [8789, 18,961]) in the mid-1990s, increased to a peak of approximately 22,525 individuals (95% CI: [13,959, 31,090]) in 2002, and slowly declined to approximately 15,368 individuals (95% CI: [9,222, 21,514]) in 2010 (Figure 4a). The decline in estimated abundance corresponded with an increase in estimated hunter effort between 2001 and 2007 from 76,457 hunter-trips to 80,017 hunter-trips, a decrease in hunter success and a decrease in productivity (Figure 4a, Table 3). Fall abundance appeared to track the rescaled fall archer indices well until approximately 2005, when estimated abundance appeared to indicate a population that was declining more slowly than the archer index would suggest.

An estimated poult-to-hen ratio provided another index available for comparison with estimates arising from the model fit. To compare this index with a data estimate, we plotted the estimated spring juvenile male turkey abundance in year *i* alongside a rescaled version of the estimated poult-to-hen ratio from late spring/early summer of year 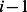 (Figure 4b). This is because the poult-to-hen ratio is obtained when turkey are approximately 1–3 months old, whereas the earliest spring abundance available from the model fit is juveniles that are roughly 1 year old and, therefore, there is a lag of approximately 9 to 11 months. Perfect concordance of the two estimates is not expected, because 1) the poult-to-hen ratio includes both males and females, and 2) it is possible (likely) that interannual fluctuations are observed in the percent of male turkeys surviving from age 1–3 months to age 12 months. In addition, it is possible that males and females survive at different rates, which would provide another source of error in the attempt to match the poult-to-hen ratio with the juvenile male abundance. Despite these limitations, results indicated a high degree of concordance between the two estimates Figure (4b). The sample correlation between these two estimates was quite high, at *p = *0.80. This suggested that increased abundance may be due to conditions which provided for an increased recruitment rate.

Parameter estimates indicated that male turkey survival was approximately 58% (95% CI: [29%, 86%]) over the course of one year, and that adult male turkeys were harvested at a rate of approximately 40% (95% CI: [29%, 52%]), while juvenile male turkeys were harvested at approximately 11% (95% CI: [5%, 17%]) at the mean level of hunter effort, 70,500 hunter trips. Interannual variation in the relationship between hunter effort and harvest vulnerability appeared to be higher for adults (0.25, 95% CI: [0.12, 0.39]) than for juveniles (0.18, 95% CI: [0.01, 0.36]), although both had wide confidence intervals.

Overall, male turkey abundance in the Ozarks East appeared to be at sustainable levels; the population is estimated to be slightly larger in 2010 than it was at the beginning of the reconstruction, in 1996. Total annual abundance, however, is estimated to have increased in the late 1990s, and then declined over the early 2000s as increased hunting pressure was exerted and productivity declined. Natural survival (for which only a single parameter could be fitted for both age classes) as well as harvest rates (estimated separately for each age class) appeared to be within expectations for similar populations from a recent study [23]. Confidence intervals around total annual abundance as well as parameters defining the demographic processes were wide. Possible reasons for wide confidence intervals include the influence of the random effects on harvest probabilities and survival parameters, low availability of cohort information, and the models themselves as well as the estimation technique employed here. Our simulation studies also generated confidence interval estimates that were relatively wide; Figures 2–3 only indicate percent coverage.

A more extensive dataset may permit the same model to be augmented with data regarding female members of the population, which may permit examination of a stock-recruit relationship, which could incorporate extrinsic factors related to recruitment of poults, such as spring rainfall. In addition, radiotelemetry studies of survival would provide an auxiliary data source with which to investigate estimating separate natural survival parameters for juveniles and adults.

## Discussion

The analysis of age-at-harvest data for small game animals presents a number of challenges in addition to those presented by large game data. Adult age classes are typically not able to be separated by age easily, so only two age classes may be available. In addition to low cohort information, small game animals tend to be impacted by extrinsic factors related to habitat status and quality, as well as environmental factors such as temperature and rainfall. Many such factors may be incorporated as covariates in the analysis (although this simulation study did not examine it) through the parameter transformations of (5), but many factors will remain unmeasured or measured with error. In addition, the functional relationship between the extrinsic factors affecting a particular demographic process (such as survival, harvest, or reproduction) is typically unknown. Despite the low cohort information, the simulation studies presented above indicate that the extension to mixed-effects statistical population reconstruction models and second-stage Horvitz-Thompson-type abundance estimates described here provide successful estimates of animal abundance, with respect to accuracy and precision of maximum likelihood estimates. Estimation accuracy and precision were generally improved with the greater amount of auxiliary data simulated. Clearly, specific cases will warrant unique considerations and additional simulation studies, such as gender-specific reconstructions, different types and amounts of available data, and availability of measured extrinsic factors that are known or hypothesized to influence population dynamics. It appears that the auxiliary catch-effort likelihood of Skalski et al. [4] (Equation 3) induces some bias in the total annual abundance estimates for the Horvitz-Thompson models *HT_FE_* and *HT_RE_* and it is therefore recommended to exclude it with these new mixed-effects models employing the Horvitz-Thompson estimator. This auxiliary likelihood component was found to be necessary to provide reliable fits for the fixed-effects absolute-recruit abundance model *AA_FE_* during simulation studies, as its exclusion led to frequent inability to maximize the log-likelihood. Bias for the model of Broms et al. [7], *AA_FE_* was estimated to be low in simulation studies, although confidence interval coverage was very low when unmeasured environmental stochasticity was simulated.

The Missouri wild turkey population reconstruction example demonstrated the utility of statistical population reconstruction models in estimating the complete suite of demographic information, including harvest probability, natural survival probability, and age-class abundance including recruit abundance. Models of this nature could be improved, however, with additional telemetry or other studies which would provide a data source to inform age-specific survival probabilities would likely improve estimates.

The models we presented in this paper considered only random effects over time. However, these models can be extended to include both temporal and spatial random effects. Applications, for example, may include the joint analysis of harvest data from adjoining game management areas. Neighboring management regions might share common survival or harvest vulnerability processes that, when analyzed jointly over time and locale, might improve the precision and understanding of local population trends.

In contrast to previously established statistical population reconstruction models for small game, random-effects models combined with second-stage, Horvitz-Thompson-type abundance estimators have proven to be capable of providing more successful process parameter and abundance estimates for statistical population reconstructions, and have also been demonstrated to be robust to some deviations from standard model assumptions. Statistical population reconstruction models enable the practitioner to associate environmental and temporal variation with demographic processes of survival and harvest, while producing realistic estimates of animal abundance as well as estimates of vital rates. These new models are very flexible, and may be employed in a wide variety of wildlife stock assessment scenarios, with a wide variety of auxiliary supporting data. Therefore, these new models are recommended to be used in statistical population reconstruction of harvested small game populations.
